# Integrated analysis of single-cell RNA sequencing and bulk RNA data reveals gene regulatory networks and targets in dilated cardiomyopathy

**DOI:** 10.1038/s41598-024-64693-2

**Published:** 2024-06-17

**Authors:** Min Zhang, Xin Zhang, Jiayin Niu, Cuncun Hua, Pengfei Liu, Guangzhen Zhong

**Affiliations:** 1 Department of Research Ward, Beijing Chao-Yang Hospital, Capital Medical University, 8 Gongren Tiyuchang Nanlu, Chaoyang District, Beijing, 100020 China; 2 Department of Urology, Beijing Chao-Yang Hospital, Capital Medical University, Beijing, China; 3 Heart Center, Beijing Chao-Yang Hospital, Capital Medical University, Beijing, China

**Keywords:** Dilated cardiomyopathy, Gene regulatory networks, Single‑cell sequencing, Regulon, Targets, Cardiovascular biology, Genomics

## Abstract

Dilated cardiomyopathy (DCM) is a common cause of heart failure, thromboembolism, arrhythmias, and sudden cardiac death. The quality of life and long-term survival rates of patients with dilated DCM have greatly improved in recent decades. Nevertheless, the clinical prognosis for DCM patients remains unfavorable. The primary driving factors underlying the pathogenesis of DCM remain incompletely understood. The present study aimed to identify driving factors underlying the pathogenesis of DCM from the perspective of gene regulatory networks. Single-cell RNA sequencing data and bulk RNA data were obtained from the Gene Expression Omnibus (GEO) database. Differential gene analysis, single-cell genomics analysis, and functional enrichment analysis were conducted using R software. The construction of Gene Regulatory Networks was performed using Python. We used the pySCENIC method to analyze the single-cell data and identified 401 regulons. Through variance decomposition, we selected 19 regulons that showed significant responsiveness to DCM. Next, we employed the ssGSEA method to assess regulons in two bulk RNA datasets. Significant statistical differences were observed in 9 and 13 regulons in each dataset. By intersecting these differentiated regulons and identifying shared targets that appeared at least twice, we successfully pinpointed three differentially expressed targets across both datasets. In this study, we assessed and identified 19 gene regulatory networks that were responsive to the disease. Furthermore, we validated these networks using two bulk RNA datasets of DCM. The elucidation of dysregulated regulons and targets (CDKN1A, SAT1, ZFP36) enhances the molecular understanding of DCM, aiding in the development of tailored therapies for patients.

## Introduction

Dilated cardiomyopathy (DCM) is a progressive myocardial disease characterized by the enlargement of the heart chambers and impaired systolic function. It is a common cause of heart failure, thromboembolism, arrhythmias, and sudden cardiac death^1,2^. Over the past few decades, significant progress has greatly improved the quality of life and long-term survival rates of patients suffering from DCM, such as early diagnosis, utilization of advanced heart failure medications^3^. Nevertheless, the clinical prognosis for patients with dilated cardiomyopathy (DCM) remains unfavorable, with a mortality rate of about 25–30% at 1 year and a survival rate of approximately 50% at 5 years^4,5^. Although scientists have identified various etiological factors for dilated DCM, including genetic mutations, infections, toxic exposures, inflammation, et al.^6,7^, the primary driving factors underlying the pathogenesis of DCM remain incompletely understood. Previous studies have indicated that the maintenance of cellular heterogeneity and the development of diseases involve the collaborative actions of multiple transcripts, with transcription factors believed to play a central role. Various transcription factors have been shown to be involved in the mechanisms underlying the development of dilated cardiomyopathy, and these factors may exert coordinated regulation at different stages of dilated cardiomyopathy differentiation^2^.

In recent years, the development of single-cell RNA sequencing (scRNA-seq) has provided new possibilities for studying cellular and gene expression. Compared to traditional bulk population sequencing, scRNA-seq enables transcriptomic analysis at the level of individual cells, unveiling transcriptional differences between different cell types and allowing the identification and characterization of cell populations or functional states that may be involved in disease. Gene regulatory networks (GRNs) are used to study the complex interactions and relationships between genes and their regulatory elements, such as transcription factors and target genes. These networks provide a framework for understanding how genes are regulated and coordinated to control various biological processes, including development, cellular differentiation, and response to environmental changes. Single-cell GRNs are an extension of traditional gene regulatory networks that specifically focus on characterizing the regulatory relationships between genes at the single-cell level, contributing to the identification of master regulators, signaling pathways, and gene modules that are critical for disease initiation or therapeutic response, and enhancing our understanding of disease mechanisms^8^.

In this study, we utilized pySECNIC to assess and identify disease-responsive gene regulatory networks and validated them in two bulk RNA datasets of DCM. By elucidating the dysregulated regulons (TF-Target gene pairs) and their associated biological pathways as well as possible targets, it contributes to a more comprehensive understanding of the molecular processes underlying the disease and helps to develop personalized therapeutic strategies for DCM patients.

## Methods

### Data collection and differentially expressed genes screening

DCM gene expression data was collected from the Gene Expression Omnibus (GEO) database (https://www.ncbi.nlm.nih.gov/geo/). We chose two datasets with sample sizes larger than 100—GSE5406^9^ and GSE57338^10^. We used "limma" R package for screening differentially expressed genes (DEGs). DEGs were filtered using a threshold of |log2(fold change [FC])|> 0.5 and a false discovery rate (FDR) < 0.05.

### ScRNA-seq data processing

We downloaded the single-cell datasets GSE109816^11^ and GSE121893^11^ from GEO database (https://www.ncbi.nlm.nih.gov/geo/). We conducted single-cell transcriptome analysis with R and processed the samples using the R package Seurat (version 4.3.0.1)^12^ Cells expressing < 200 or > 5000 genes were filtered out. After the data was normalized (NormalizeData function), the top 2000 highly variable genes were detected using the "FindVariableFeatures" function. Then, the data was scaled using the "ScaleData" function. Principal component analysis (PCA) was performed using the variable features (RunPCA function) to reduce the dimensionality of scRNA-seq data. "Harmony" R package^13^ was applied to integrate 18 individual datasets. UMAP embeddings were generated (RunUMAP function) using "harmony" reduction. Similarly, graph-based clustering was performed using "harmony" reduction and cell clustering resolution was set to 0.7. The FindAllMarkers function was employed to identify signature genes for each cluster, and cellular identities were subsequently annotated the cells based on the related literature references and the CellMarker2.0 database^14^. Differential expression analysis comparing two groups of cells was conducted using the FindMarkers function (logfc.threshold = 0.25, min.pct = 0.1). The Wilcoxon rank-sum test was utilized for the differential analysis, and the Benjamini–Hochberg method was applied to control the false discovery rate.

### Gene regulatory networks and hub TFs

We performed dimensionality reduction and clustering analysis on the data using the "Seurat" package with a resolution parameter of 50. Subsequently, we filtered out metacells that had a minimum cell count greater than 10. As a result, we obtained 425 normal cells and 166 DCM cells as the retained metacells. pySCENIC package (Python Single-Cell rEgulatory Network Inference and Clustering)^15^ was used to construct a regulatory network connecting DCM-associated targets with transcription factors. Firstly, the GRNboost2 algorithm was used to infer the gene–gene co-expression relationships between transcription factors (TF) and their potential targets (pyscenic grn). Secondly, the regulon prediction step was performed (pyscenic ctx). Each regulon contained one TF and its target genes enriched for the motifs of the TF. The motif annotation databases were downloaded from https://resources.aertslab.org/cistarget. Lastly, we analyzed the single-cell transcriptomic data using the AUCell algorithm to assess the activity scores of each regulon (pyscenic aucell). Utilizing the matrix of regulon activity scores (RAS) derived from pySCENIC analysis, we employed the UMAP algorithm to facilitate dimensionality reduction. Subsequently, we visualized the first two RAS-UMAP dimensions based on cell clusters or groups. We performed a heatmap visualization analysis on the presumed regulon using the ComplexHeatmap R package. The philentropy package was used to identify cell type-specific regulons, and the ggrepel package was utilized for visualization purposes.

### Regulon modules analysis based on connection specificity index (CSI) matrix

In this study, we utilized the context-specific CSI method to uncover and analyze regulon modules, which quantify the connections between transcription factors (TFs) and their respective target genes^16^. We began by computing the Pearson correlation coefficient (PCC) for activity scores among various regulon pairs,  and transformed the RAS matrix to calculate these correlations. We then defined the CSI between any two regulons, A and B, by calculating the proportion of other regulons with which neither A nor B shares a higher PCC than that shared between A and B themselves. This approach allowed us to assess the specificity of connections between individual TFs. Subsequently, we employed hierarchical clustering with Euclidean distance on the CSI matrix to discern distinct regulon modules, which was visualized using the "ggplot2" package. We singled out the regulon module that displayed the most notable inter-group variances for further investigation. These differences could potentially hold significant implications for understanding the development and progression of DCM.

### Variance decomposition

To assess the contribution of cell types and the groups to regulon variation, we applied a linear mixed model (LMM). Regulon expression was modeled as a function of cell types and the groups (considered as random factors)^17^. The LMM was implemented in the R package lme4^18^. The restricted maximum likelihood estimators for the random effects of cell types, groups, and residual variance were normalized by their sum to give the variance components. We selected regulons whose variance proportions were predominantly explained by groups.

### Functional enrichment analysis

Single-sample gene set enrichment analysis (ssGSEA) was performed using the GSVA R package to compare the enrichment scores of significant regulons between the normal and DCM groups in the bulk RNA dataset. The stat_compare_means function from the ggpubr package was utilized to perform the statistical tests. Subsequently, the visualization was carried out using the ggboxplot function from the ggpubr package.

By intersecting the target genes of the 19 regulons with the differentially expressed genes derived from two bulk RNA datasets, we identified the specific DEGs associated with the regulons. Then, we utilized the "clusterProfiler" R package to conduct Gene ontology (GO) and the Kyoto Encyclopedia of Genes and Genomes (KEGG) analysis based on these DEGs^19,20^.

### Diagnostic performances analysis

For the assessment of diagnostic efficacy of the hub targets, we utilized the "roc" function from the "pROC" package to perform binary classification analysis. This approach allowed us to compute the area under the ROC curve (AUC) and estimate its 95% confidence interval using a non-parametric bootstrap method. Cross-validation was implemented through bootstrap resampling to confirm the reliability of our AUC estimates.

### Statistical analysis

R software (version 4.2.1; Rstudio, Boston, MA) was used for all statistical analyses. Student's t-test or Mann–Whitney U test was used to compare two groups of continuous variables. For multiple comparisons, parametric data were analyzed using one-way analysis of variance (ANOVA), while non-parametric data were assessed using Kruskal–Wallis tests. The scCustomize package assists in visualizing data. A significance level of P < 0.05 was considered statistically significant.

## Results

### Expression of key genes in the scRNA-seq dataset

**Figure 1:**
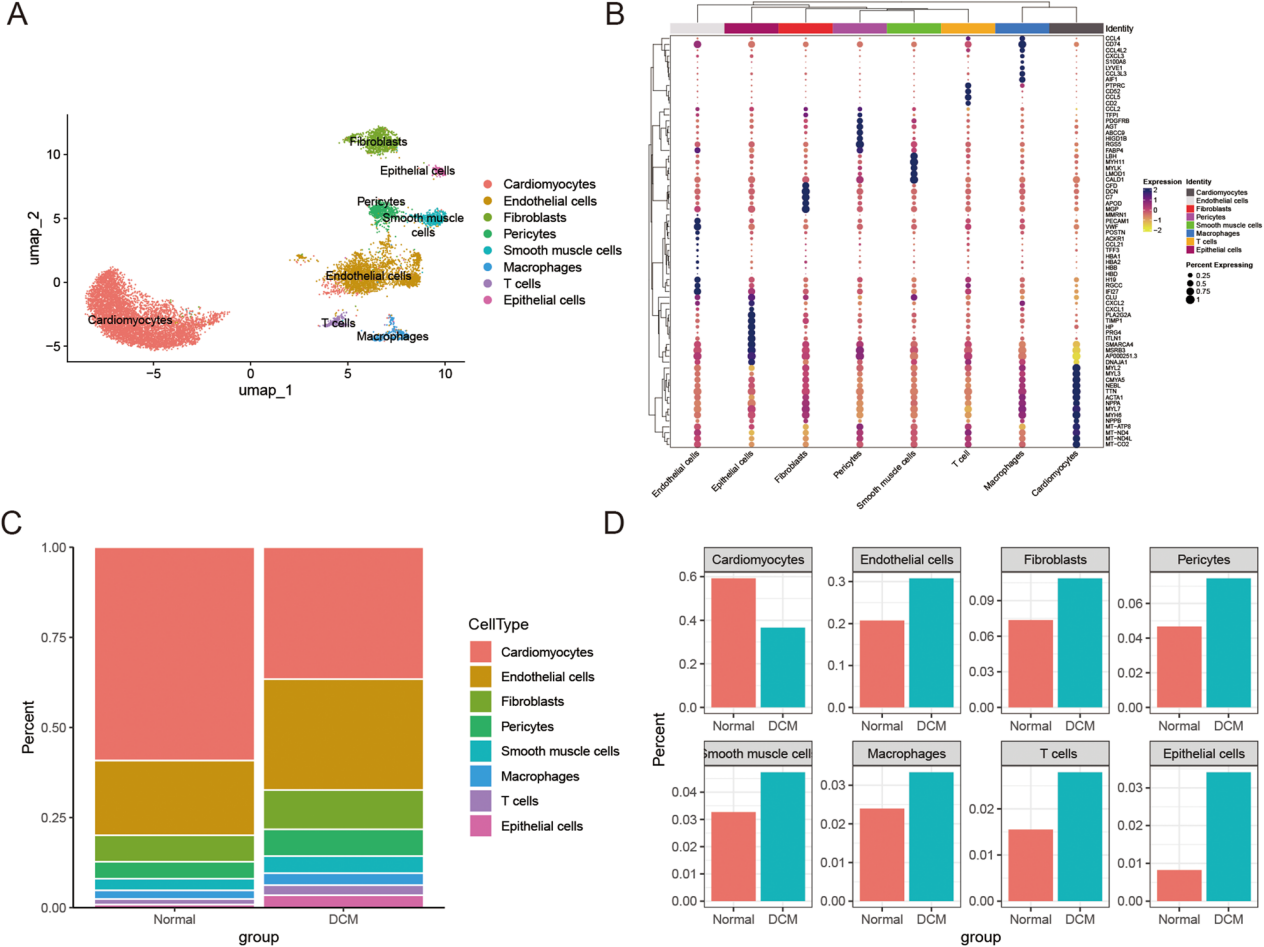
ScRNA-seq analysis of myocardial tissue. (**A**) UMAP plot shows 11,930 cells isolated from normal and DCM patients. Cell clusters are color coded. (**B**) Dot plot showing the selected top DEGs in each UMAP cluster. Columns represent cell clusters, and rows represent genes. (**C**) Specific cell abundance in different sample types. (**D**) The intergroup variations of specific cell abundance.

The scRNA-seq datasets consisted of 14 healthy donors and 4 DCM patients (Table 1). Figure S1A,B displayed Uniform Manifold Approximation and Projection (UMAP) plots before and after the integration of the 18 samples. We clustered all cells into eight subsets based on known marker genes (Fig. 1A), including cardiomyocytes (TTN, MYH6), endothelial cells (VWF, PECAM1, CDH5), fibroblasts (DCN, LUM), pericytes (ABCC9, PDGFRB), smooth muscle cells (MYH11, CALD1, SPARC), macrophages (CD163, MRC1), T cells (CD2, CD3D), epithelial cells (PRG4, ITLN1). Differential analysis was conducted on these subgroups to identify the most significant differentially expressed genes within each cluster, which are visualized in Fig. 1B. Next, we analyzed the proportions of specific cells in different sample types (Fig. 1C,D). In the normal group, cardiomyocytes were the most abundant, constituting 59.2% of the total cell population. The remaining cells included endothelial cells, which accounted for 20.7%, fibroblasts at 7.3%, and pericytes at 4.7%. Other cell types comprised less than 4% each. In contrast, in the DCM group, the proportion of cardiomyocytes was decreased to 36.3%. The percentages for endothelial cells and fibroblasts were higher compared to the normal group, at 30.1% and 10.9%, respectively. A detailed breakdown of the total cell counts and proportions for each cell type is presented in Table S1.

**Table 1:** Basic information of datasets involved in the study.

Datasets	Normal	DCM	Tissue	Platforms	Applications	References (PMID)
GSE5406	16	86	Myocardium	GPL96	Validation	16952980
GSE57338	136	82	Myocardium	GPL11532	Validation	25528681
GSE109816	12	0	Myocardium	GPL18573	Single-cell RNA sequencing analysis	31915373
GSE121893	2	4	Myocardium	GPL18573	Single-cell RNA sequencing analysis	31915373

### Identification of regulon using pySCENIC

**Figure 2:**
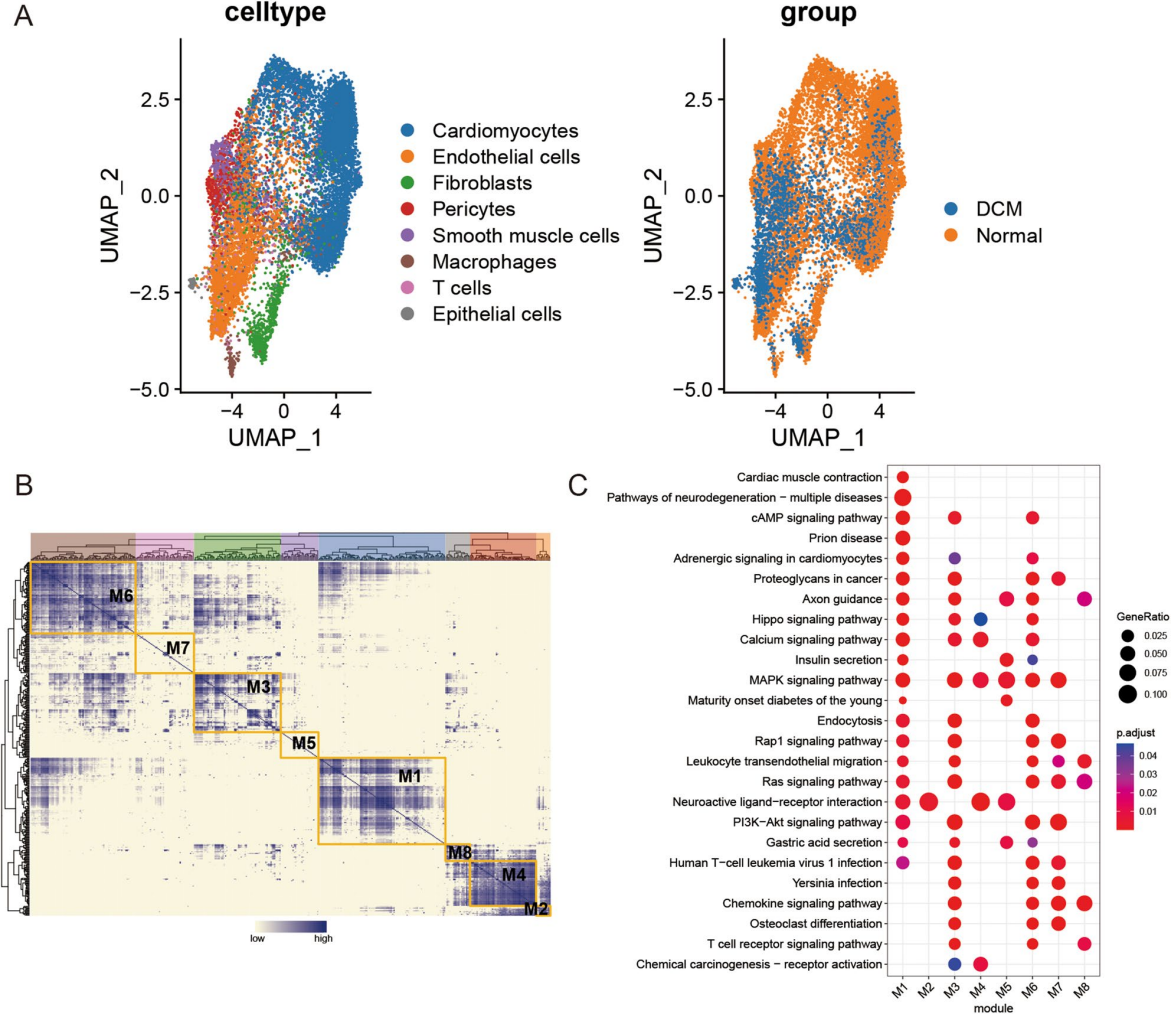
Identification of regulon using pySCENIC. (**A**) Differential regulon activity scores between the Normal and DCM groups. (**B**) Determination of the regulon modules. Columns represent regulons, and rows represent cells. The color bar, indicating "low" and "high", reflects the degree of correlation in expression levels between these regulons across the various cell samples. (**C**) Enrichment analysis of the targets in each module.

We derived a RAS matrix using pySCENIC analysis. Then, the UMAP algorithm was utilized to conduct an unsupervised clustering analysis. Specifically, the Normal group displayed considerable enrichment in cardiac muscle cells, while the DCM group exhibited a significant regulon enrichment in endothelial cells (Fig. 2A).

Based on the CSI between 401 regulons (TF-Target gene pairs) identified by pySCENIC, cells were clustered into eight modules (M1–M8) (Fig. 2B). The average activity of regulons within each module is depicted in Fig. S2. Enrichment analysis of the targets in each module revealed that M1 was primarily enriched in Cardiac muscle contraction, cAMP signaling pathway, Adrenergic signaling in cardiomyocytes, et al. Meanwhile, M3 exhibited enrichment in PI3K-Akt signaling pathway, Ras signaling pathway, etc. While M6 was predominantly enriched in pathways related to PI3K-Akt signaling pathway, MAPK signaling pathway, cAMP signaling pathway, etc. (Fig. 2C).

### Variance decomposition

**Figure 3:**
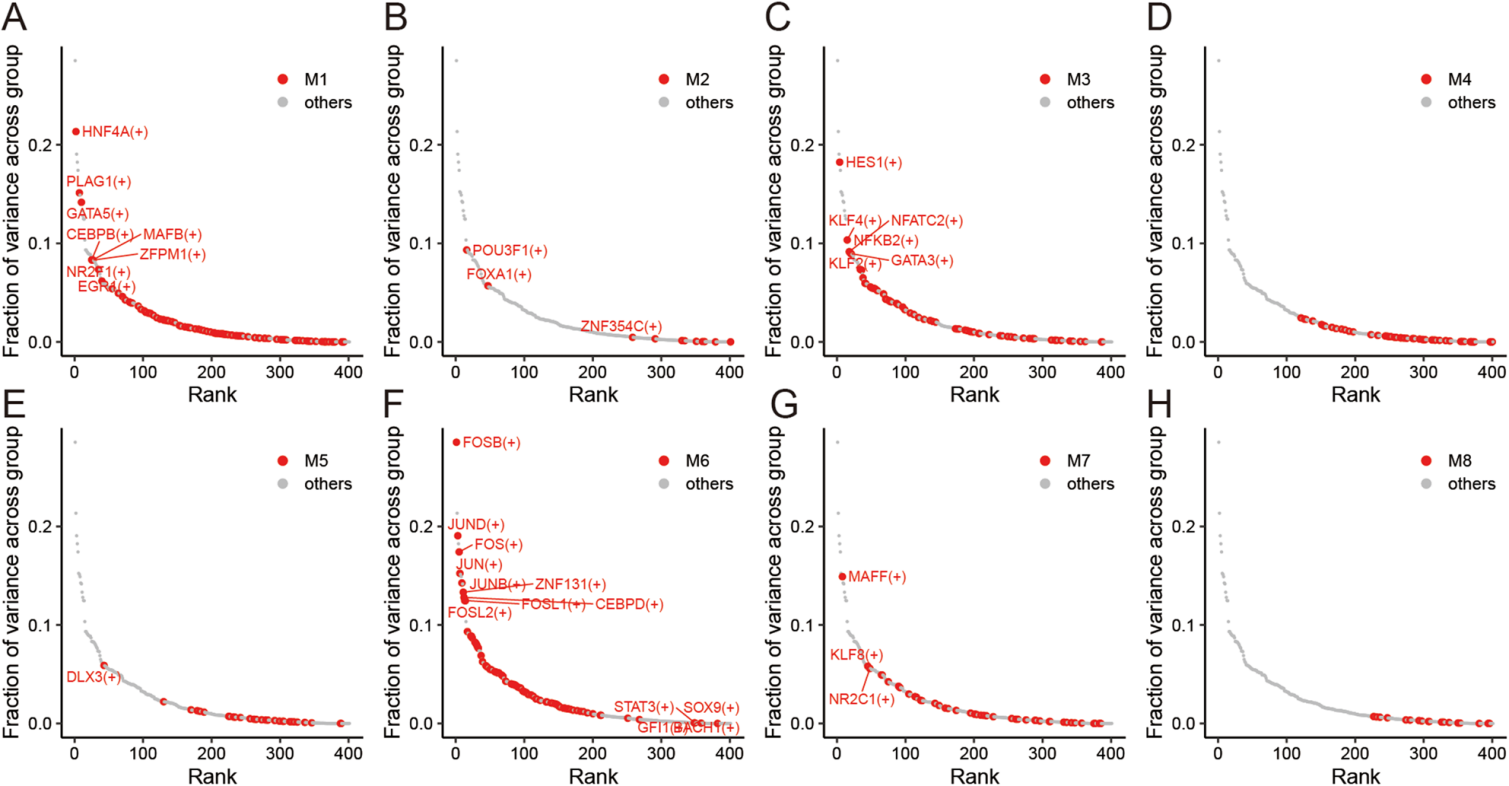
Variance decomposition of eight modules.

We conducted variance decomposition to further explore the meaningful regulons within each module. As shown in Fig. 3, the regulons above the inflection point (Points with slope less than − 1) contribute to the grouping. The specific variance decomposition results for each regulon can be found in Table S2. There are four regulons in M1, namely HNF4A(+), PLAG1(+), GATA5(+), and CEBPB(+). M3 consists of five regulons, namely HES1(+), KLF4(+), NFATC2(+), NFKB2(+) and GATA3(+). M6 includes nine regulons, namely FOSB(+), JUND(+), FOS(+), JUN(+), JUNB(+), ZNF131(+), CEBPD(+), FOSL1(+) and FOSL2(+). M7 contains one regulon, namely MAFF(+). The expression levels of these 19 regulons in the Normal and DCM groups are shown in Fig. S3.

Additionally, we isolated normal cells and investigated cell type-specific regulons within this population. The findings are visualized in Fig. S4. Interestingly, the regulons MAFF and NFKB2 were found to be both responsive to the disease and specific to cell types. The representative regulons for each cell type are displayed in Fig. S5A–H.

### Screening DEGs of bulk RNA datasets

**Figure 4:**
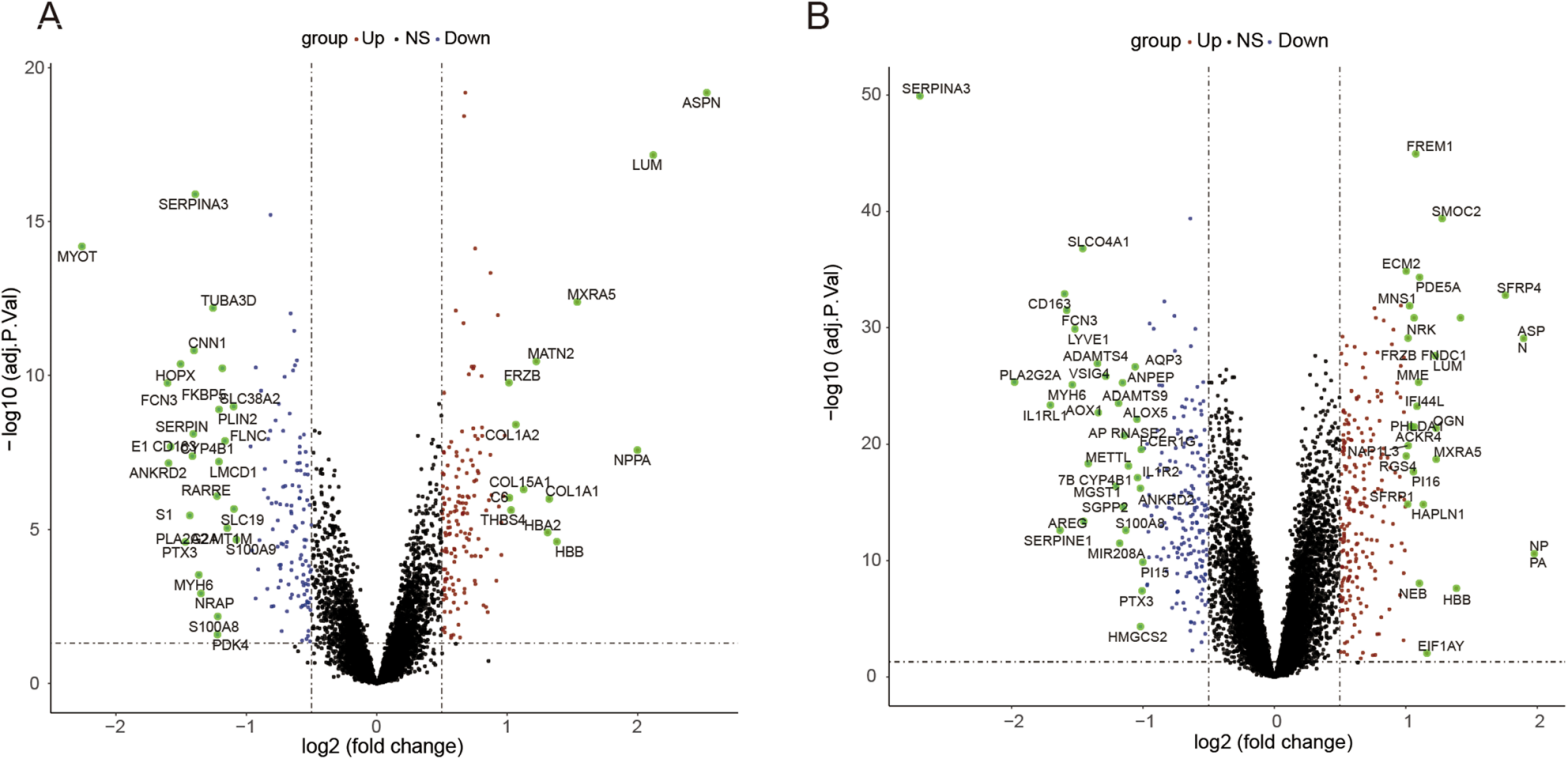
Volcano plots of two bulk RNA datasets. (**A**) Volcano plots of GSE5406. (**B**) Volcano plots of GSE57338.

Differential expression analysis was performed on two bulk RNA datasets. The number of differentially expressed genes obtained for each dataset based on the filtering criteria is presented in Table 1. Volcano plots illustrating the differentially expressed genes obtained from the two datasets are shown in Fig. 4A, B.

### Functional enrichment analysis

**Figure 5:**
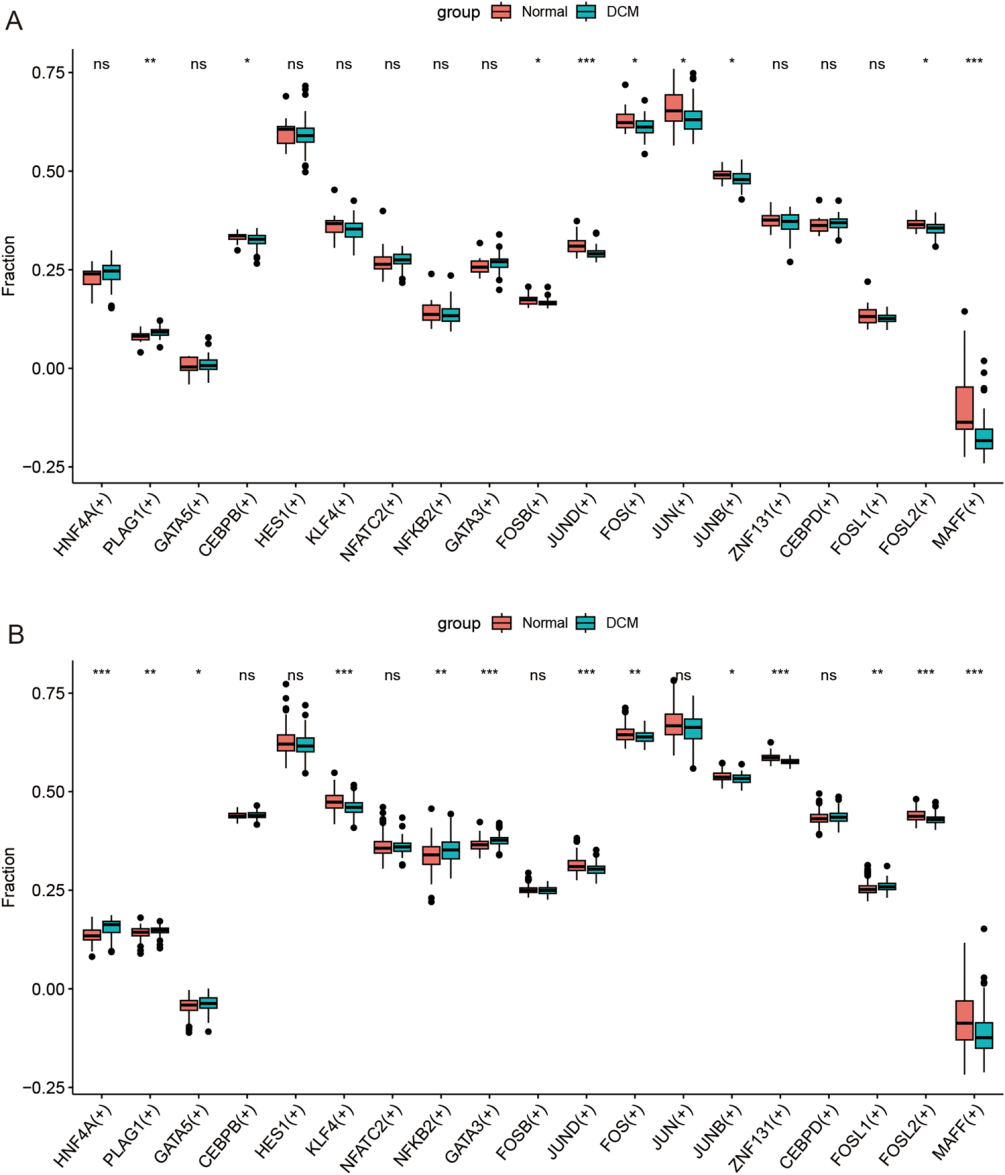
Comparison of the ssGSEA scores of 19 regulons between the Normal and DCM groups. The "Fraction" on the y-axis represents the ssGSEA scores, which measure the enrichment level of regulons within each sample. These box plots include the median (represented by the central line of the box), interquartile range (represented by the edges of the box), and potential outliers (indicated by points). (**A**) GSE5406 dataset (16 Normal samples and 86 DCM samples). (**B**) GSE57338 dataset (136 Normal samples and 82 DCM samples). *P < 0.05, **P < 0.01, ***P < 0.001, *ns* no significance.

We extracted 19 significant regulons obtained from the single-cell datasets as gene sets and performed enrichment analysis using the ssGSEA method on two bulk RNA datasets. The results are presented in Fig. 5A,B. There were 9 and 13 differentiated regulons in the normal and DCM groups of the two datasets, respectively.

Moreover, enrichment analysis of DEGs associated with the regulons in the two datasets revealed that GO annotations were mainly focused on cardiac tissue-related processes, including muscle contraction, cardiac chamber morphogenesis, etc. The KEGG analysis results revealed that the DEGs associated with the regulons in both datasets were significantly associated with the FoxO signaling pathway, PI3K-Akt signaling pathway, and p53 signaling pathway, which was consistent with the enrichment analysis results obtained from the single-cell dataset (Fig. S6A,B).

### Identifying the hub targets

**Figure 6:**
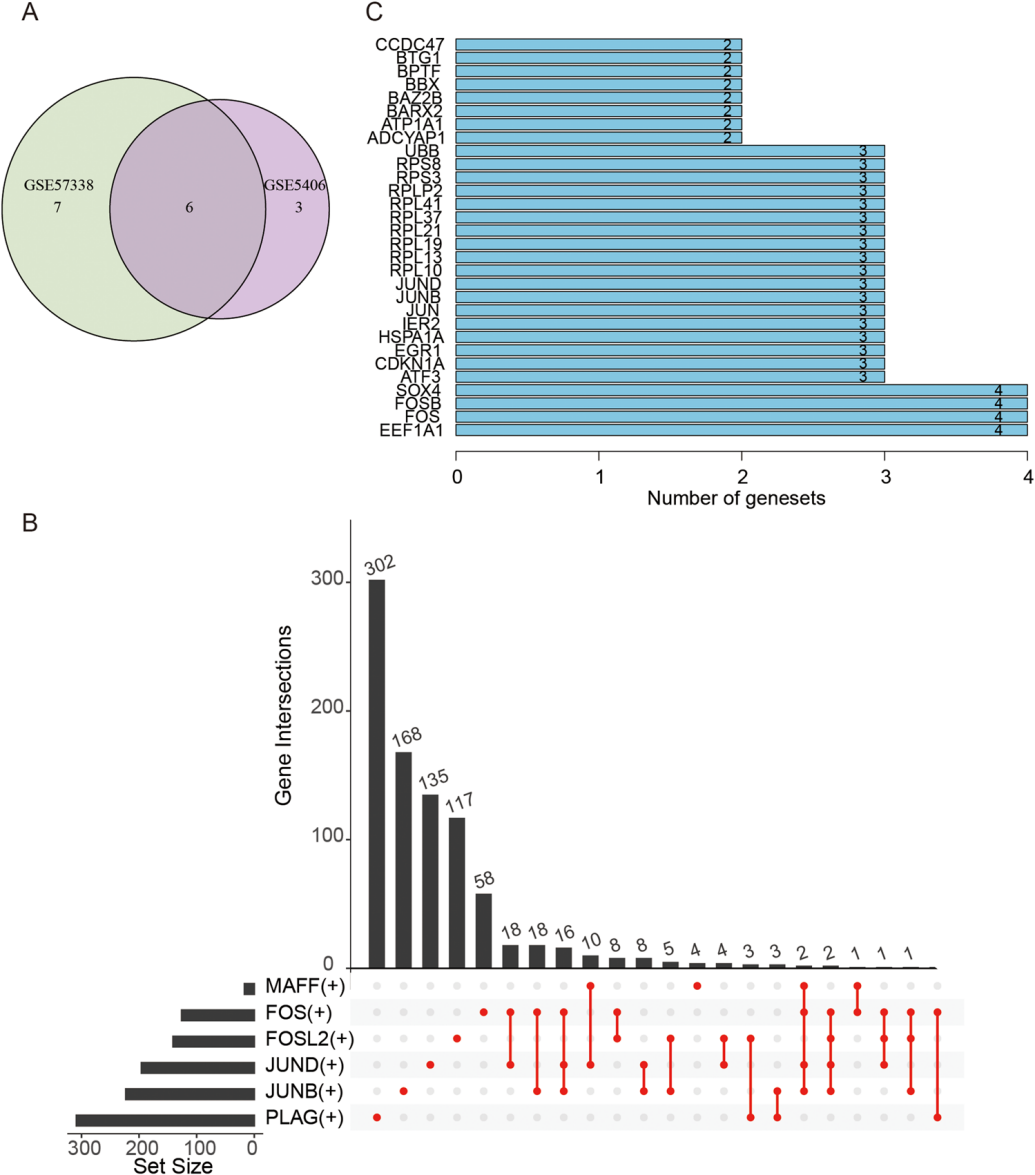
Venn plots. (**A**) Intersecting the differentiated regulons from the two bulk RNA datasets. (**B**) An upset plot visualizes the targets that occur at least twice. (**C**) The barplot visualizes the top 30 targets.

We observed six differentiated regulons across two datasets by intersecting the two bulk RNA datasets, as depicted in a Venn diagram (Fig. 6A). Six regulons exhibited consistent trends in both datasets. Specifically, we identified five regulons [JUND(+), FOS(+), JUNB(+), FOSL2(+) and MAFF(+)] that exhibited a decrease in expression levels in DCM, while the PLAG1(+) regulon showed an upregulation in DCM. Next, we explored the targets that appeared at least twice in six regulons, totaling 101 targets (Fig. 6B,C), the specific targets were listed in Table S3. Subsequently, we intersected these 101 targets with the DEGs from two datasets, resulting in eight and four differentially expressed targets, respectively. Finally, after retaking the intersection, we obtained three differentially expressed targets: CDKN1A, SAT1, and ZFP36. CDKN1A is under the regulation of three regulons, namely FOS(+), JUND(+), and JUNB(+). SAT1 is regulated by two regulons, namely FOS(+) and FOSL2(+). ZFP36 is targeted by FOS(+) and JUNB(+).

### Expression and diagnostic efficacy of hub targets

**Figure 7:**
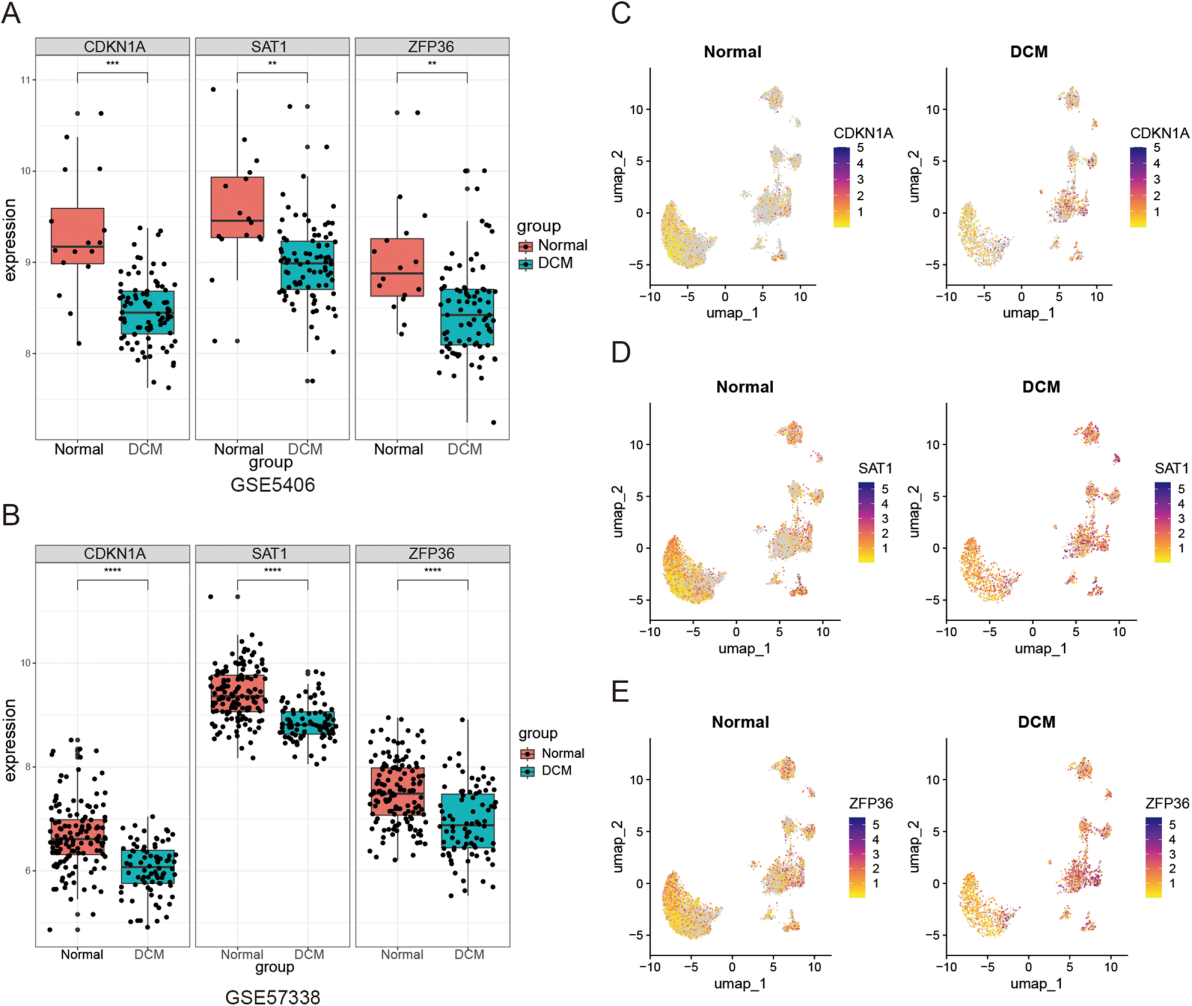
Target expression levels. Targets expression levels in GSE5406 (**A**) and GSE57338 (**B**). Expression levels of CDKN1A (**C**), SAT1 (**D**) and ZFP36 (**E**) in single-cell datasets. **P < 0.01, ***P < 0.001, ****P < 0.0001.

Finally, we explored the expression levels and diagnostic efficacy of hub targets in two datasets. In both datasets, the expression levels of three targets (CDKN1A, SAT1, and ZFP36) were consistently higher in the normal group compared to the DCM group (P < 0.05) (Fig. 7A, B). The AUC values for the three targets in datasets GSE5406 and GSE57338 were 0.873, 0.805, 0.77 and 0.788, 0.817, 0.731, respectively (Fig. S7). Furthermore, our analysis of scRNA-seq data (Fig. 7C–E) revealed that these three genes exhibit high expression, specifically in endothelial cells of DCM.

## Discussion

In our analysis, we uncovered key regulatory networks in DCM by integrating single-cell RNA sequencing data and applying computational methods. We identified a set of 19 disease-responsive regulons and, through further analysis, pinpointed three differentially expressed targets across bulk RNA datasets. These findings shed light on the molecular underpinnings of DCM and set the stage for future investigations.

Prior to pySCENIC analysis, we created metacells, which corresponded to partitions of single-cell data into disjoint homogeneous groups of highly similar cells followed by aggregation of their profiles^21^. This method effectively reduces the computational burden by decreasing the number of cells analyzed from 11,930 to 591, without compromising the signal-to-noise ratio. By aggregating cells into metacells, we were able to diminish the impact of technical noise and enhance biological signal in sparse single-cell genomics data. This strategy not only improves the computational efficiency of the pySCENIC analysis but also ensures that the resulting gene regulatory network inferences are more robust and biologically meaningful. In this study, we applied a variance decomposition algorithm to quantify the contributions of disease and cell type as factors influencing regulons. This allowed us to identify regulons that are responsive to the disease. In addition to disease-responsive GRNs, we also investigated cell type-specific GRNs. We systematically analyzed regulons that may play a role in the occurrence and development of DCM. During the validation process, we chose bulk RNA datasets with sample sizes greater than 100 to enhance the reliability of the results. In the specific target selection process, we focused on targets that appeared at least twice in six regulons, further indicating their involvement in DCM.

DCM is typically associated with cardiomyocytes loss. In this research, the DCM group showed a decrease in the proportion of myocardial cells while the proportion of other cell types increased. Myocardial fibrosis is the main pathological change in DCM, characterized by excessive proliferation of cardiac interstitial fibroblasts, collagen deposition, and abnormal distribution in the cardiac interstitium^22^. During the progression of DCM, endothelial cells injury can trigger an inflammatory response and release various chemical signaling molecules, including profibrotic factors, inflammatory cytokines, and growth factors, etc., which promote cardiac fibrosis and affect tissue repair and regeneration of the heart^23,24^. Additionally, endothelial cells can promote cardiac fibrosis through endothelial-to-mesenchymal transition (EndMT)^25^. Furthermore, endothelial cells participate in angiogenesis (neovascularization) and blood supply to myocardial tissue. In this study, the expression of three targets in endothelial cells was increased in the DCM group, suggesting that these three molecules play a role through endothelial cells in DCM.

The CDKN1A gene, coding for the potent cyclin-dependent kinase inhibitor CDKN1A/p21, was the main induced gene in p53-mediated cell cycle arrest^26^. The CDKN1A protein can directly interact and inhibit CDK complexes regulating the cell cycle progression at the G1 phase^27^. In a mouse Langendorff model of hypoxia-reoxygenation myocardial injury, Rev-Erbα gene deletion or antagonist treatment protected cardiomyocytes from cell death through an increase in the expression of CDKN1a/p21^28^. Yücel et al. proposed that p21 inhibition can induce cardiac cell cycle activity in cultured myocardial cells of mice, rats, and humans^29^. A GWAS study identified an association between genetic variations in the CDKN1A gene and the risk of heart failure^30^.

The SAT1 protein, belonging to the acetyltransferase family, catalyzes the acetylation of spermidine and spermine. Previous studies have shown that depletion of SAT1 in cells results in changes in gene expression programs, including cell cycle regulation and DNA repair^31^. Zhong et al. identified that SAT1 is typically expressed more in the left ventricle of the heart than in the left atrium, and is associated with the development of heart failure through scRNA-seq  Data^32^.

The ZFP36 gene, which codes for the protein commonly known as tristetraprolin or TTP, binds to the AU-rich region in the 3'-UTR of mRNA to negatively regulate the production of protein from mRNA transcripts^33^. ZFP36 is involved in multiple cellular processes and plays a crucial role in the cellular response to cytokine and growth factor stimulation, as well as in the regulation of gene expression. It functions in mRNA decay and metabolism processes, thereby influencing mRNA stability and degradation rate^33^. Previous studies have demonstrated that ZFP36 modulates inflammatory activities^34,35^. Zhang et al. reported that enhanced expression of ZFP36 in aortic endothelial cells might reduce vascular inflammation through direct binding to target cytokine mRNAs^36^. A meta-analysis of single-cell RNA sequencing targeting multiple species revealed that ZFP36 regulates human coronary artery endothelial cell proliferation in ischemic hearts and determined that VEGF-C administration in vivo enhances clonal expansion of the cardiac vascular after myocardial infarction^37^. Our research indicates that CDKN1A, SAT1, and ZFP36 share overlapping upstream transcription factors, which are in fact members of the AP-1 family, playing an important role in regulating cellular proliferation, differentiation, and biological response processes^38^. AP-1 mainly includes members of the Fos family (such as c-Fos, FosB, FOSL1, and FOSL2) and the Jun family (such as c-Jun, JunB, and JunD), among others. In our study, genes regulated by AP-1, namely CDKN1A, SAT1, and ZFP36, were found to be downregulated in DCM (dilated cardiomyopathy) myocardium compared to normal myocardium. Renata Windak et al., using pressure overload-induced cardiac hypertrophy in mice and targeted deletion of Jun in cardiomyocytes, demonstrated that c-jun was required for adaptive cardiac hypertrophy^39^. Denise Hilfiker-Kleiner et al. also showed in their research that the lack of JunD promoted pressure overload-induced apoptosis, hypertrophic growth, and angiogenesis in the heart^40^. However, Michael A. Burke et al., using a PLN (phospholamban) mutation mouse model at different stages of DCM progression, found that the JunB gene was upregulated in DCM myocardium^41^. The possible reason is that in the PLN gene mutation DCM mouse model, the TGFβ pathway is significantly activated, regulating the upregulation of JunB. This disease model may differ from the pathological process of human DCM, hence the direction of change in JunB is also different.

In the present study, we employed the single-cell GRN to screen for disease-responsive regulons and validated them in bulk RNA datasets. However, there are certain limitations to our research. Firstly, the targets we identified as highly expressed in the DCM group of the single-cell dataset showed downregulation in the bulk RNA data. We hypothesize that this discrepancy is due to the association of these three genes with cell types other than cardiomyocytes. However, the bulk RNA samples cannot differentiate between cell types and primarily consist of cardiomyocytes, which decreases the expression levels of these three genes at the overall level. Our finding is consistent with previous studies that have reported a decrease in their expression, specifically in cardiomyocytes of DCM^42,43^. Secondly, the biological functions of these three genes need further validation in in vitro and in vivo models. While we observed their association with disease response in the single-cell dataset, it is crucial to obtain additional experimental evidence to understand their roles in the progression of cardiac diseases fully.

## Conclusion

In this study, we identified 19 gene regulatory networks that are responsive to the disease in single-cell data. Furthermore, we validated these regulons using two bulk RNA datasets and identified three differentially expressed targets (CDKN1A, SAT1, and ZFP36). Overall, our study provides valuable insights into the regulatory mechanisms underlying disease response and helps to develop personalized therapeutic strategies for DCM patients.

### Supplementary Information

Supplementary Figure 1.

Supplementary Figure 2.

Supplementary Figure 3.

Supplementary Figure 4.

Supplementary Figure 5.

Supplementary Figure 6.

Supplementary Figure 7.

Supplementary Legends.

Supplementary Table 1.

Supplementary Table 2.

Supplementary Table 3.

## Data Availability

The datasets presented in this study can be found in the GEO database: https://www.ncbi.nlm.nih.gov/geo/.
