# Supplementary figures and images for: Integrated analysis of single-cell RNA sequencing and bulk RNA data reveals gene regulatory networks and targets in dilated cardiomyopathy
Source: Sci Rep. 2024 Jun 17;14:13942. doi: 10.1038/s41598-024-64693-2 (PMC11183045; doi:10.1038/s41598-024-64693-2)

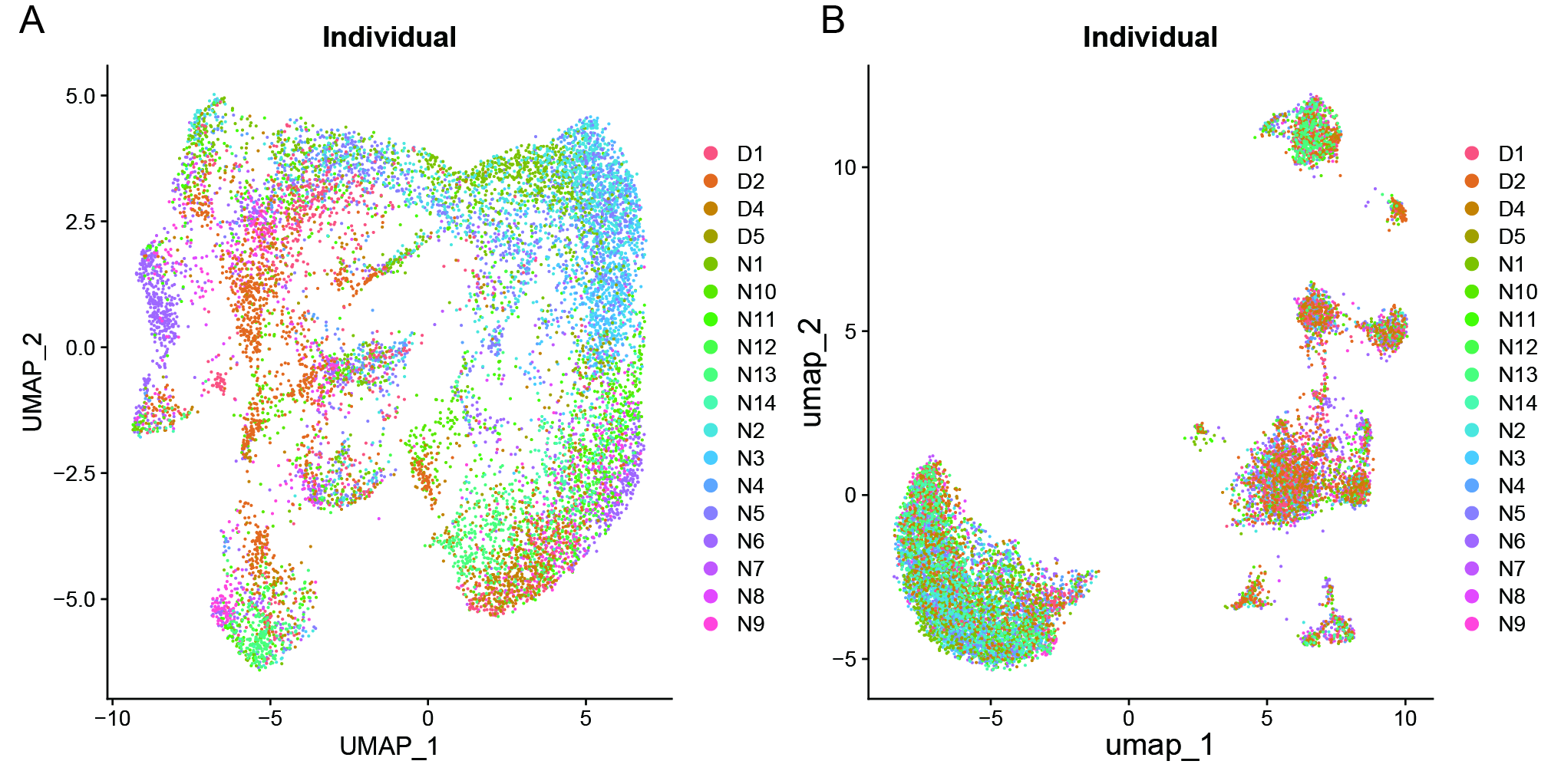

Supplement: Supplementary file 1 — Supplementary Figure 1. [file 41598_2024_64693_MOESM1_ESM.tif]

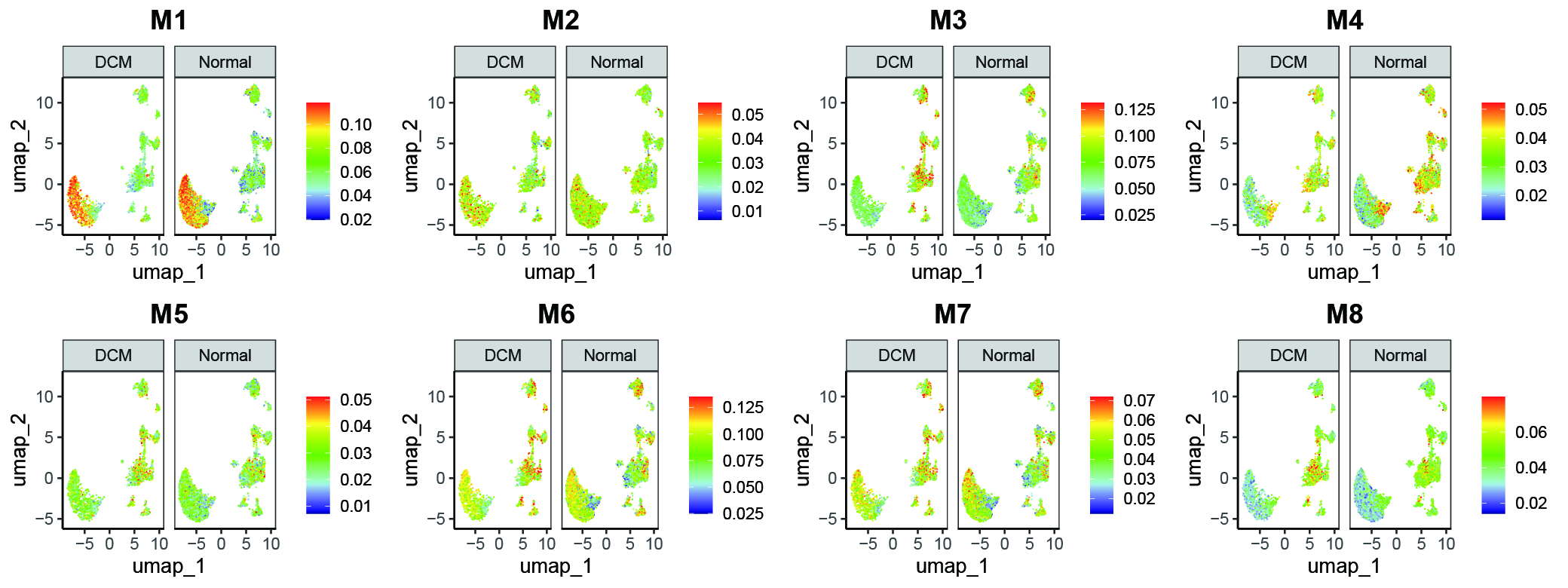

Supplement: Supplementary file 2 — Supplementary Figure 2. [file 41598_2024_64693_MOESM2_ESM.tif]

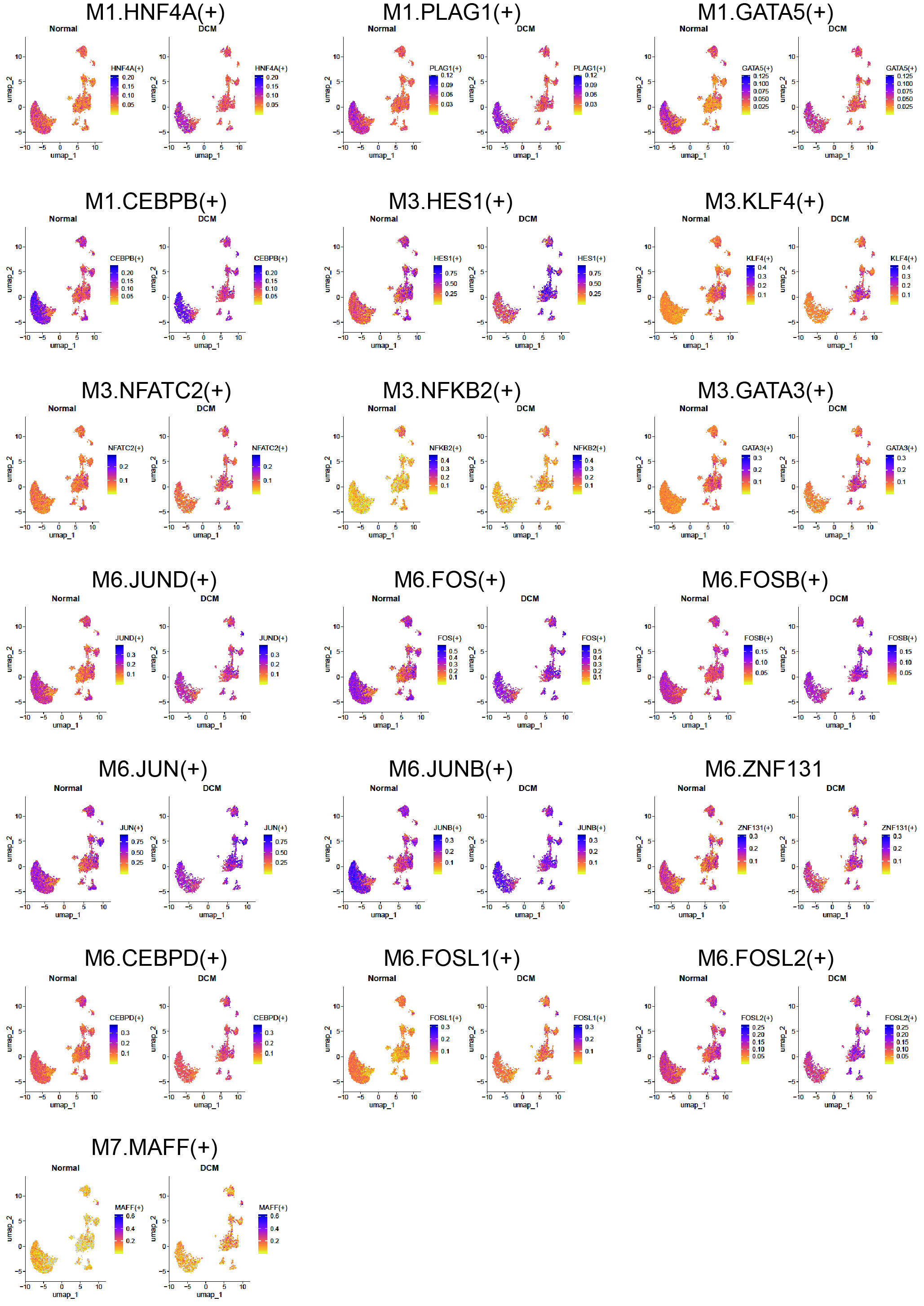

Supplement: Supplementary file 3 — Supplementary Figure 3. [file 41598_2024_64693_MOESM3_ESM.tif]

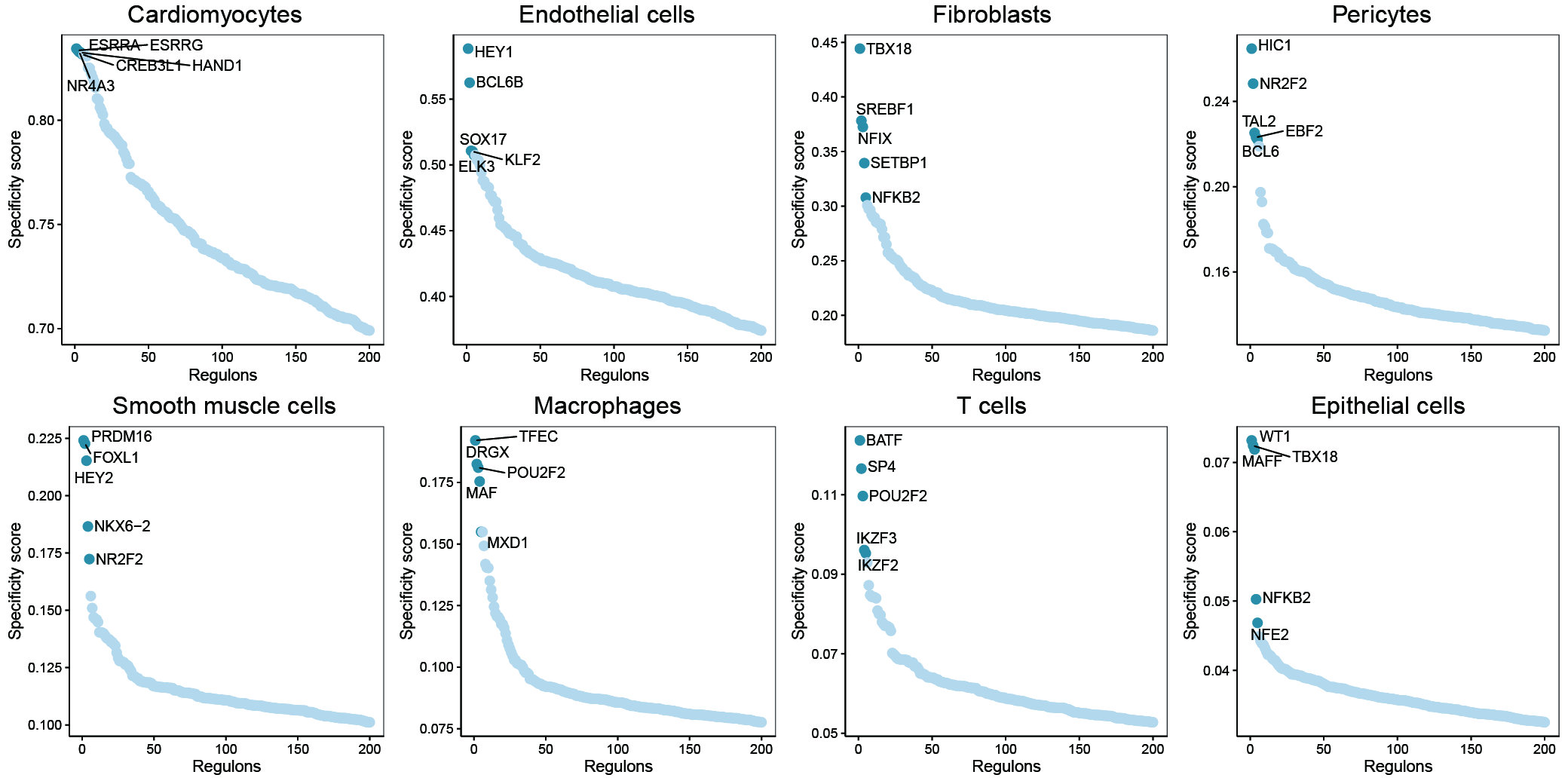

Supplement: Supplementary file 4 — Supplementary Figure 4. [file 41598_2024_64693_MOESM4_ESM.tif]

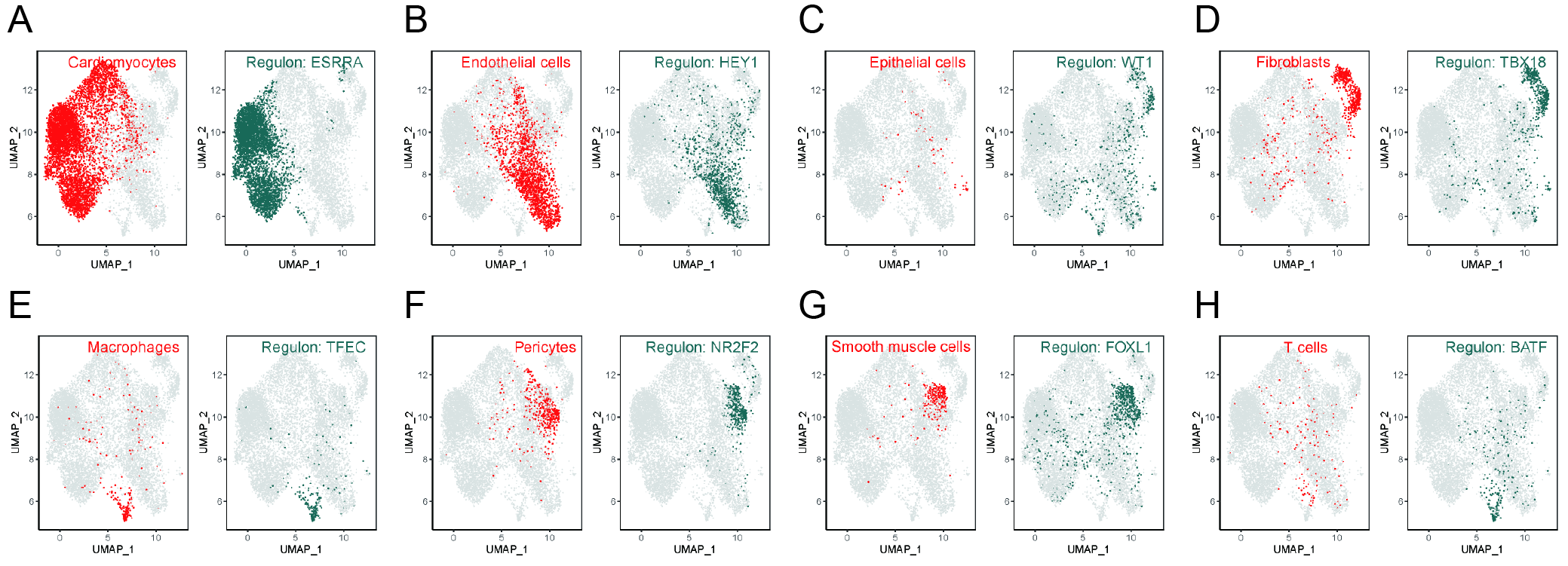

Supplement: Supplementary file 5 — Supplementary Figure 5. [file 41598_2024_64693_MOESM5_ESM.tif]

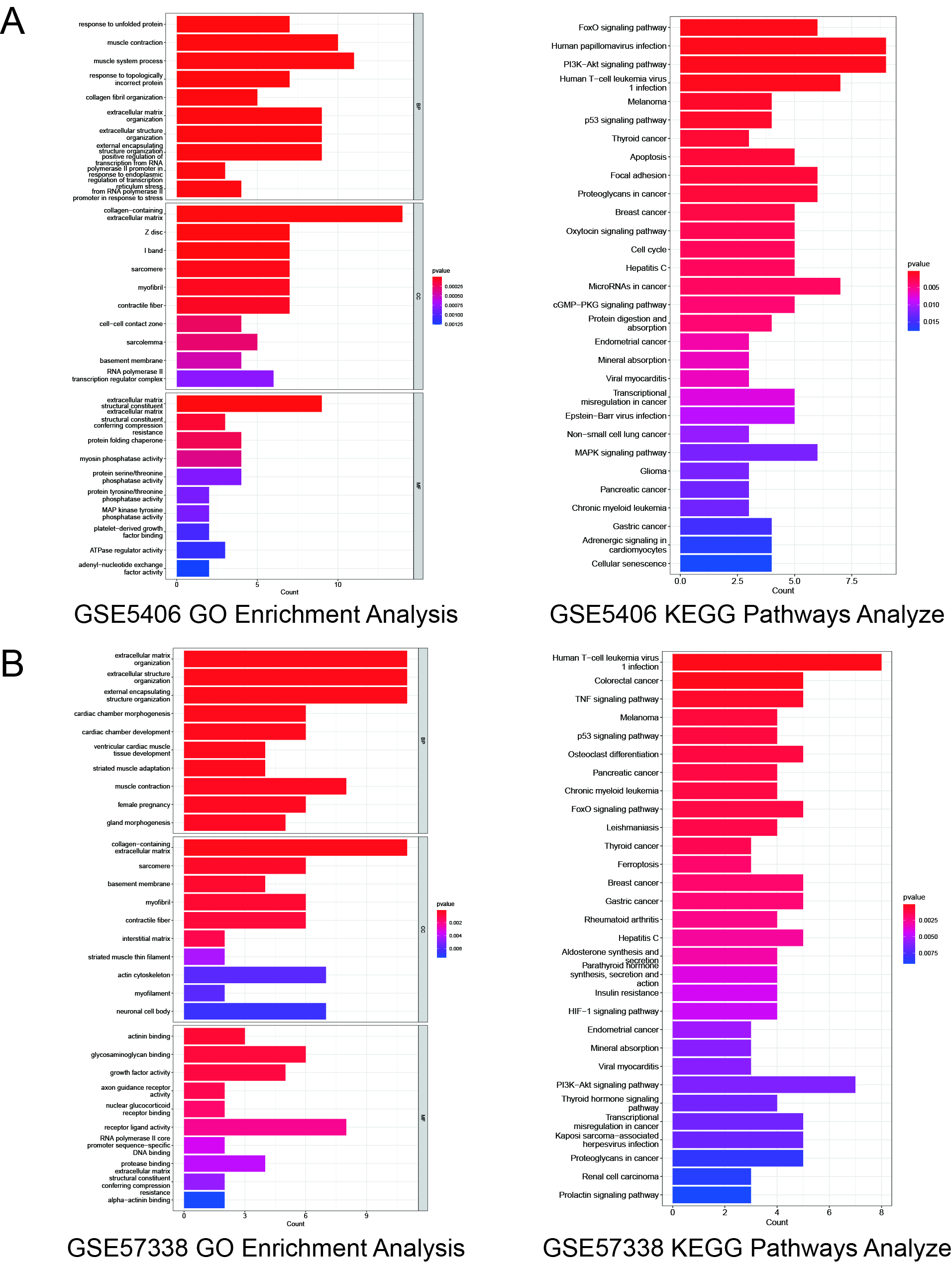

Supplement: Supplementary file 6 — Supplementary Figure 6. [file 41598_2024_64693_MOESM6_ESM.tif]

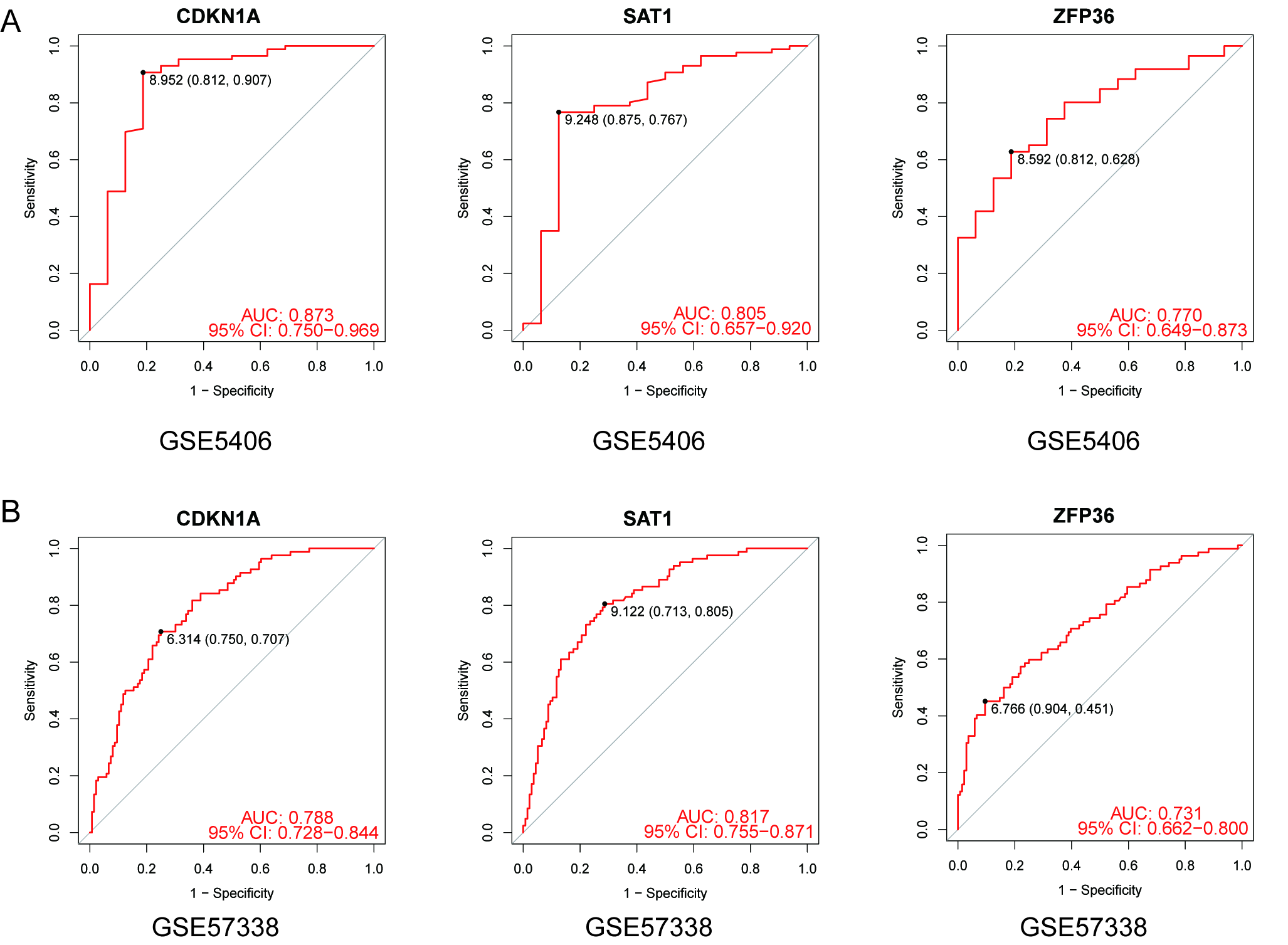

Supplement: Supplementary file 7 — Supplementary Figure 7. [file 41598_2024_64693_MOESM7_ESM.tif]
