# Supplementary material for: Integrated analysis of single-cell RNA sequencing and bulk RNA data reveals gene regulatory networks and targets in dilated cardiomyopathy
Source: Sci Rep. 2024 Jun 17;14:13942. doi: 10.1038/s41598-024-64693-2 (PMC11183045; doi:10.1038/s41598-024-64693-2)
Supplement: Supplementary file 8 — Supplementary Legends. [file 41598_2024_64693_MOESM8_ESM.docx]

Supplementary Table S1-S3 legends

Table S1 Detailed cell type-specific counts and proportions in normal and DCM groups

Table S2 Variance decomposition results for each regulon. This table details the variance decomposition analysis for each regulon, clarifying the contributions of cell type, group, and residual variance. The 'celltype' column represents the proportion of variance ascribed to differences among cell types, while the 'group' column denotes the variance associated with the distinction between normal and DCM samples. The 'residual' column accounts for the variance not explained by cell type or group.

Table S3 Comprehensive list of specific target genes and their corresponding counts

Supplementary Figure Legends

Figure S1A-B UMAP plots before and after integration of the 18 samples.

Figure S2 The average activity of each module.

Figure S3 The expression levels of these 19 regulons.

Figure S4 Cell type-specific regulons within normal cells. The blue dots represent the meaningful regulons in each cell type.

Figure S5 The representative significant regulons for each cell type. The left panel represents the distribution of cell types, while the right panel represents the distribution of cell type-specific regulons.

Figure S6 Functional Enrichment Analysis. (A) GO and KEGG analyses of GSE5406. (B) GO and KEGG analyses of GSE57338.

Figure S7 Diagnostic efficacy of hub genes in the prediction of DCM in GSE5406 (A) and GSE57338 (B) cohort.
